# Effects of age on postoperative oral function in older adults with oral squamous cell carcinoma and its cutoff values: a cross-sectional study

**DOI:** 10.1038/s41598-025-91017-9

**Published:** 2025-03-21

**Authors:** Tatsuhito Kotani, Yuhei Matsuda, Mayu Takeda, Reon Morioka, Yukiho Shimamura, Rie Osako-Sonoyama, Hiroto Tatsumi, Masako Fujioka-Kobayashi, Takahiro Kanno

**Affiliations:** 1 Department of Oral and Maxillofacial Surgery, Shimane University Faculty of Medicine, 89-1, Enya-Cho, Izumo, Shimane 693-8501 Japan; 2 Department of Oral and Maxillofacial Surgery, Masuda Red Cross Hospital, 103-1, Otoyoshi-cho, Masuda, Shimane 698-0003 Japan

**Keywords:** Oral squamous cell carcinoma, Age, Oral function, Postoperative oral dysfunction, Cutoff, Cancer, Oral cancer, Surgical oncology

## Abstract

This study aimed to investigate the influence of age on postoperative oral function in older adults with oral squamous cell carcinoma and its cutoffs. 102 patients (74 males; 28 females) with oral squamous cell carcinoma were treated according to the National Comprehensive Cancer Network guidelines at the Department of Oral and Maxillofacial Surgery, Shimane University Hospital, between September 2019 and March 2023 were included. Their mean age was 69.6 years. Background data were obtained from the medical records, and oral function measurements were performed the day before discharge. Final analysis was performed using the receiver operating characteristic (ROC) curve and propensity score. The primary tumor site was the tongue in 45 (44.1%), gingiva in 41 (40.2%), and other sites in 16 (15.7%) patients. ROC analysis showed a cutoff age of 75 years for occlusal force. The propensity score method showed that the odds ratio of age was 4.32 (confidence interval: 1.48 − 12.55 [*P* = 0.01]) for occlusal force. Treatment age is independently associated with postoperative oral function, and the incidence of postoperative oral dysfunction is approximately four times higher for patients aged > 75 years. The development of measures for postoperative oral function recovery may be particularly necessary in older adult patients aged > 75 years.

## Introduction

According to the National Comprehensive Cancer Network (NCCN) guidelines, surgery is the first choice of treatment for oral squamous cell carcinoma (OSCC), and several modalities are added as adjuncts depending on the cancer stage^1^. In recent years, the development of immune checkpoint inhibitors and photoimmunotherapy has increased treatment options for advanced OSCC^2^. A phase 2 open-label, multicenter study of photoimmunotherapy in patients with locoregional, recurrent head and neck squamous cell carcinoma reported a median overall survival of 9.3 months^3^. Novel therapies are expected to improve survival in the future. The standard primary outcome for OSCC, as for other cancers, is the 3- or 5-year survival rate^4^. There are variations among reports, but the 5-year survival rate is approximately 50% for patients with OSCC^5^. While survival and disease-free survival rates are important for cancer treatment, Japan’s super-aged society requires a reconsideration of outcomes in the treatment of older patients with OSCC. Lohr reported that the 5Ds (death, disease, disability, dissatisfaction, and discomfort) are important for outcome measurements, and death and disease are often important for cancer treatment^6^. However, difficult outcomes such as survival rate are very important for children and older adults; however, disability, dissatisfaction, and discomfort are considered when selecting treatment as a clinical decision^7^. Therefore, soft outcome (disability, dissatisfaction, and discomfort) considerations and the definition of OSCC in older adults should be considered during the treatment of OSCC.

Japan has the world’s oldest population, and Shimane Prefecture, the field of this study, is a regional city with one of the largest aging populations in the country^8^. The number of treated older adults with OSCC has been increasing with the number of patients with OSCC. For example, Karino et al. reported the case of an 89-year-old patient with advanced tongue cancer who strongly desired treatment. The patient was selected for treatment with an emphasis on the recovery of oral function (reconstructive surgery with consideration of swallowing function using a hyoid bone suspension technique and creation of a palatal augmentation prosthesis [PAP]), as well as survival rate as outcomes of the treatment^9^. A good outcome was achieved after treatment maintaining oral intake. OSCC treatment was implemented, but consideration was given to soft outcomes after treatment, such as disability (oral dysfunction and dysphagia), dissatisfaction (non-retention of a previous lifestyle), and discomfort (decreased quality of life). This case shows that survival rate is not always the primary outcome in older patients with OSCC. Klingelhöffer et al. reported a hazard ratio of 2.26 for the effect of age on tumor-related survival of 400 patients with OSCC, with a cutoff value of ≥ 65 years^10^. There have been reports on the relationship between survival rate and age, but there are no reports on the cutoff value of age related to oral function and age after treatment so far in the literature. The dilemma of treatment outcomes for older adults with OSCC is not limited to Japan, but it is a problem that will be faced in the future by developed countries facing super-aging societies.

A common understanding of the definition of oral function has emerged^11^. In Japan, oral hypofunction was registered as a new disease by the National Health Insurance in 2018, and seven unified oral function measurements were determined^12^. In 2021, Matsuda et al. reported that six methods of measuring oral function in patients with OSCC were applicable and defined the rapid decline in oral function after OSCC treatment as postoperative oral dysfunction in three categories (transport type, oral hygiene type, and occlusion type)^13^. It has been reported that postoperative oral dysfunction and dysphagia after OSCC treatment significantly reduce the quality of life of patients^14^. It is especially important to consider the quality of life, including maintenance of oral function, because patients with OSCC have been reported to have a higher rate of suicide after treatment than patients with other cancers^15^. In addition, primary death owing to tumors of the oral cavity or head and neck region is not only physically and psychologically but also financially detrimental because it is accompanied by changes in facial morphology; thus, local tumor control and maintenance of oral function should be considered during treatment selection^16^. Therefore, a cutoff value for the age at which oral function may be significantly compromised after OSCC treatment should be considered. This cutoff value is expected to contribute significantly to patient decision-making when considering treatment strategies. The hypothesis in this study was that older age is associated with lower oral function after OSCC treatment and a cutoff value could be used to discriminate the risk of oral functional decline after OSCC treatment.

Therefore, the purpose of this study was to examine the relationship between age and post-treatment oral function in patients with OSCC and to determine the age cutoff value.

## Patients and methods

### Patient eligibility

This single-center, cross-sectional study used a sequential sampling method. Data from patients who completed primary OSCC treatment between September 2019 and March 2023 were used in this study. The eligibility criteria were as follows: patients diagnosed with primary OSCC; those being treated at the Shimane University Hospital Oral and Maxillofacial Surgery/Oral Care Center followed by standard treatment according to the NCCN version 8.0 guidelines; those aged ≥ 20 years; and those able to complete the questionnaire. The exclusion criteria were as follows: cases in which oral function could not be measured owing to death or cognitive function decline, and cases of optional or nonstandard treatment based on the guidelines.

This study was approved by the Medical Research Ethics Committee of Shimane University Faculty of Medicine (number 4041). Written informed consent was obtained from each participant before participation in the study. Furthermore, all methods were performed in accordance with Declarations of Helsinki.

### Patient background data

The following patient background data were collected from electronic medical records: sex (male/female), age (years), primary tumor site (tongue, maxillary gingiva, mandibular gingiva, palate, oral floor, and buccal mucosa), clinical cancer stage, treatment method (surgery, surgery and radiotherapy, and surgery and chemoradiotherapy), presence of neck dissection, presence of reconstructive surgery, and body mass index (kg/m^2^).

### Oral function measurement

**Fig. 1:**
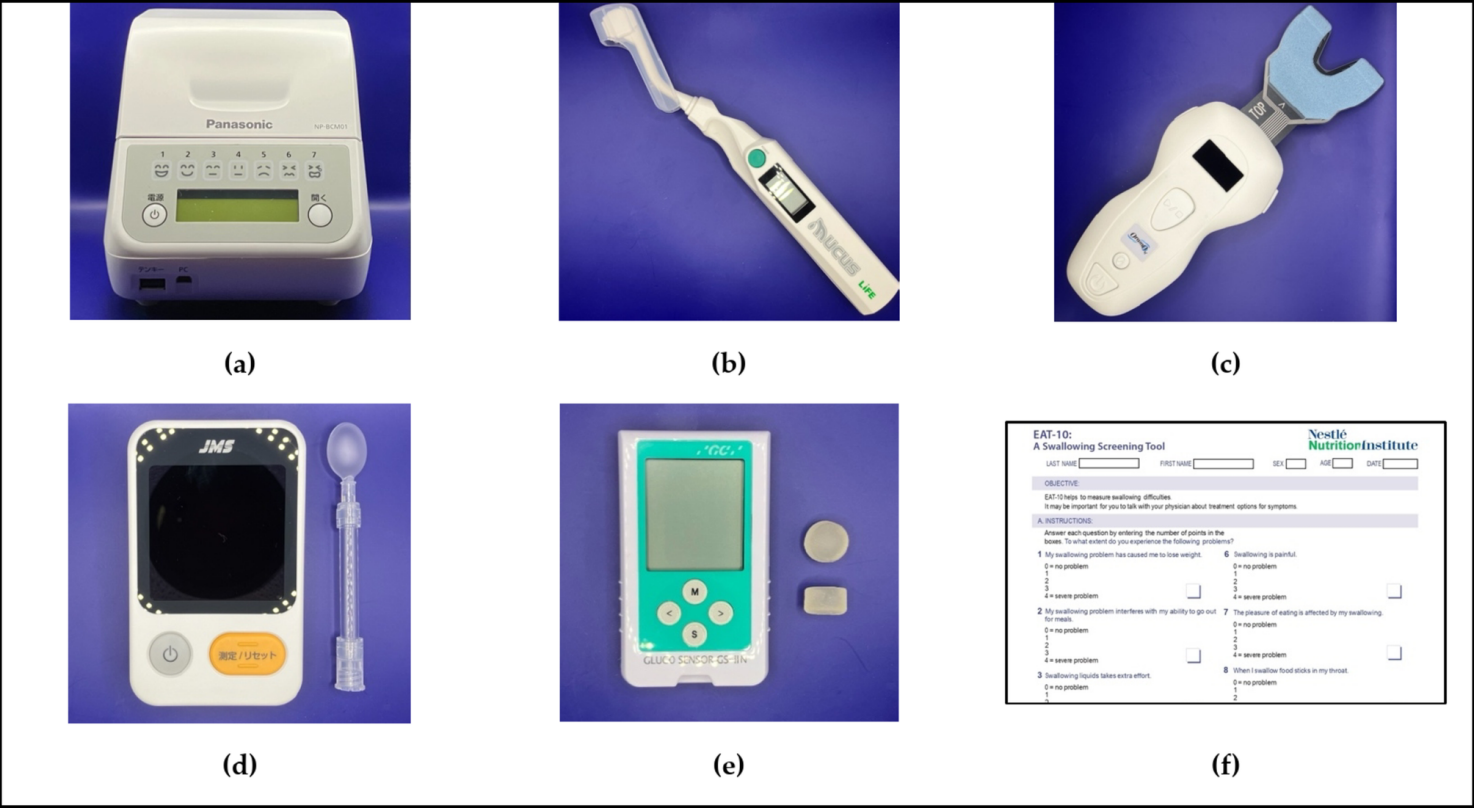
Devices used for oral function measurement: (a) Bacterial counter (b) Mucus (c) Dental Prescale Occluzer (d) TPM-01 (e) Gluco Sensor GS-II (f) EAT-10. EAT-10: Eating Assessment Tool-10.

For oral function measurements, data were collected for six of the seven items recommended by the Japanese Society of Gerodontology, which are diagnostic indicators of postoperative oral dysfunction^11,13^. The number of microorganisms was measured by collecting samples from the center of the tongue dorsum using a rapid oral detection apparatus (bacterial counter; Panasonic Healthcare, Tokyo, Japan; Fig. 1-a). Oral dryness was measured using an oral moisture checker (Mucus; Life, Saitama, Japan; Fig. 1-b), and the median of three measurements on the dorsum of the tongue was used as the data. The occlusal force was measured using a pressure-sensitive paper (Dental Prescale Occluzer; GC, Tokyo, Japan; Fig. 1-c) by clenching for 3 s at the intercuspal position. If the participant had a denture, the occlusal force was measured with the denture in place. Tongue pressure was measured at the center of the dorsum of the tongue using a tongue pressure-measuring instrument (TPM-01; JMS, Hiroshima, Japan; Fig. 1-d). Masticatory function was measured using a masticatory ability testing system (Gluco Sensor GS-II; GC, Tokyo, Japan; Fig. 1-e). Swallowing function was assessed using a 10-question questionnaire (Eating Assessment Tool [EAT]-10; Fig. 1-f) with a 5-point Likert scale (0 = no problem; 4 = severe problem) developed by Belafsky in 200,820. The EAT-10 has a maximum total score of 40, with higher scores indicating poor swallowing function, as proposed by Matsuda et al.^13^. All oral function measurements were taken immediately before the patient’s return to society after primary treatment, according to the standard treatment of the NCCN version 8.0. Measurements were taken with dentures and PAP whenever possible.

### Statistical analysis

The Shapiro–Wilk test was used to confirm the normality of the distribution of the data. Continuous data are described as means and standard deviations, and categorical data are described as numbers and percentages. Pearson’s correlation coefficients were calculated to determine the association between continuous data, and scatter plots were created to visualize the relationships. Multiple linear regression analysis (forced entry method) was conducted, considering confounding factors. Analysis of the receiver operating characteristic (ROC) curves and the area under the curve (AUC) was used to examine the sensitivity and specificity of each oral function measurement to detect the high-risk age for postoperative oral dysfunction. Each patient was assigned an estimated propensity score, which was the predicted probability of postoperative oral dysfunction based on the cutoff score reported in a previous study^13^. Finally, multivariable logistic regressions were also performed by applying propensity scores to adjust for group differences using the inverse probability of treatment weighting (IPTW)^17^.

Statistical analyses were performed using IBM SPSS Statistics for Windows, version 27. (IBM Corp., Armonk, NY, USA). Two-tailed p-values were calculated for all analyses, and the alpha level of significance was set at *P* < 0.05.

## Results

### Demographic data and clinical characteristics

In total, 102 patients with OSCC were enrolled: 74 (72.5%) males and 28 (27.5%) females with a mean age of 69.6 years. The background factors and information about cancer and its treatment are shown in Table 1.

**Table 1:** Demographic characteristics of patients with OSCC (*N* = 102).

Items	Categories	*n* (%) or mean [SD]
Sex	Male	74 (72.5)
	Female	28 (27.5)
Age (years)		69.6 [13.6]
Primary tumor site	Tongue	45 (44.1)
	Maxillary gingiva	19 (18.6)
	Mandibular gingiva	22 (21.6)
	Palate	3 (2.9)
	Oral floor	5 (4.9)
	Buccal mucosa	8 (7.8)
Clinical cancer stage	I	26 (25.5)
	II	12 (11.8)
	III	14 (13.7)
	IV	50 (49.0)
Treatment	Surgery	53 (52.0)
	Surgery and radiotherapy	45 (44.2)
	Surgery and chemoradiotherapy	31 (30.4)
Neck dissection	(Yes)	64 (62.7)
Reconstructive surgery	(Yes)	58 (56.9)
Body mass index	(kg/m^2^)	21.5 [4.0]
Oral function measurement	Tongue pressure (kPa)	16.4 [11.6]
	Microorganisms (Grade)	3.3 [1.5]
	Oral dryness	23.2 [5.1]
	Masticatory function (mg/dL)	97.1 [77.5]
	Occlusal force (N)	313.0 [333.3]
	EAT-10	14.7 [11.4]

OSCC: oral squamous cell carcinoma, SD: standard deviation, EAT-10: Eating Assessment Tool-10.

### Relationship between age and postoperative oral function

**Fig. 2:**
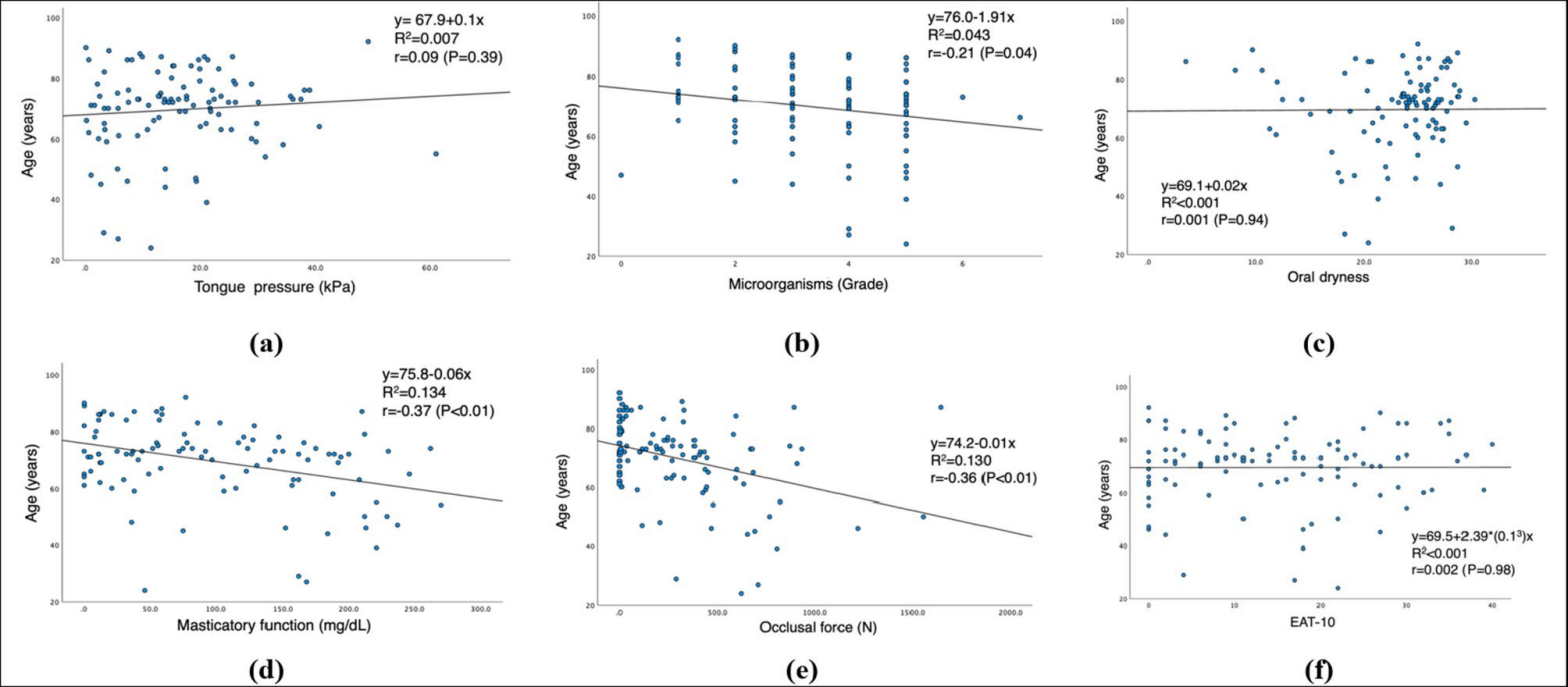
Correlation between age and each oral function measurement: (a) tongue pressure (b) microorganisms (c) oral dryness (d) masticatory function (e) occlusal force (f) EAT-10. EAT-10: Eating Assessment Tool-10.

The correlations between age and each oral function measurement are shown in Fig. 2. Microorganisms, masticatory function, and occlusal force were significantly correlated with age (*P* < 0.05).

### Correlation between age and oral function using univariate and multivariate analyses

The results of the univariate and multivariate analyses with masticatory function as the objective variable are shown in Table 2. In the univariate analysis, age (B=-2.08, *P* < 0.01), clinical cancer stage (B=-18.36, *P* < 0.01), surgery (B = 39.13, *P* = 0.01), and surgery and radiotherapy (B=-37.02, *P* = 0.02) were significantly associated with age. In multivariate analysis, only age was significantly associated with age (B=-0.37, *P* < 0.05).

**Table 2:** Univariate and multivariate analysis with masticatory function as the objective variable.

Items	Univariate					Multivariate				
	Standardized B	B	95% CI		P-value	Standardized B	B	95% CI		P-value
			Lower	Upper				Lower	Upper	
Age (years)	-0.37	-2.08	-3.14	-1.03	< 0.01*	-2.09	-0.37	-3.25	-0.93	< 0.01*
Sex	0.03	4.70	-29.57	38.97	0.79	2.25	0.01	-30.69	35.18	0.89
Primary tumor site (tongue)	0.05	7.35	-23.43	38.13	0.64	-11.38	-0.07	-45.07	22.31	0.50
Primary tumor site (others)	0.09	18.15	-21.82	58.12	0.37	17.27	0.09	-26.63	61.16	0.44
Clinical cancer stage	-0.31	-18.36	-29.68	-7.04	< 0.01*	-14.19	-0.24	-35.08	6.70	0.18
Surgery	0.25	39.13	9.51	68.75	0.01*	40.87	0.27	-52.08	133.83	0.39
Surgery and radiotherapy	-0.24	-37.02	-66.95	-7.10	0.02*	23.73	0.15	-55.48	102.94	0.55
Surgery and chemoradiotherapy	-0.18	-29.60	-62.34	3.15	0.08	-7.83	-0.05	-53.55	37.89	0.74
Neck dissection	-0.18	-28.27	-59.42	2.87	0.08	27.53	0.17	-25.55	80.61	0.31
Reconstructive surgery	-0.17	-26.89	-57.32	3.54	0.08	-15.19	-0.10	-50.34	19.95	0.39

CI: confidential interval.

The results of the univariate and multivariate analyses with occlusal force as the objective variable are shown in Table 3. In the univariate analysis, age (B=-8.83, *P* < 0.01), primary tumor site (tongue; B = 252.47, *P* < 0.01), clinical cancer stage (B=-90.78, *P* < 0.01), surgery (B = 157.57, *P* = 0.02), surgery and radiotherapy (B=-137.50, *P* = 0.04) and neck dissection (B=-173.73, *P* = 0.01) were significantly associated with age. In the multivariate analysis, age (B=-0.22, *P* = 0.03) and primary tumor site (B=-0.57, *P* = 0.002) were significantly associated with age.

**Table 3:** Univariate and multivariate analysis with occlusal force as the objective variable.

Items	Univariate					Multivariate				
	Standardized B	B	95% CI		P-value	Standardized B	B	95% CI		P-value
			Lower	Upper				Lower	Upper	
Age (years)	-0.36	-8.83	-13.37	-4.29	< 0.01*	-5.30	-0.22	-10.04	-0.57	0.03*
Sex	-0.07	-53.55	-200.63	93.53	0.47	-31.30	-10.04	-165.9	103.32	0.65
Primary tumor site (tongue)	0.38	252.47	129.76	375.18	< 0.01*	217.68	-0.57	80.00	355.37	0.002*
Primary tumor site (others)	-0.03	-24.36	-196.91	148.20	0.78	91.97	0.03	-87.42	271.36	0.31
Clinical cancer stage	-0.35	-90.78	-138.66	-42.90	< 0.01*	-66.36	-0.04	-151.72	19.00	0.13
Surgery	0.24	157.57	29.61	285.52	0.02*	180.14	-165.9	-199.78	560.06	0.35
Surgery and radiotherapy	-0.21	-137.50	-267.20	-7.80	0.04*	131.30	103.32	-192.44	455.04	0.42
Surgery and chemoradiotherapy	-0.11	-82.14	-224.29	60.00	0.25	62.84	0.65	-124.03	249.70	0.51
Neck dissection	-0.25	-173.73	-305.40	-42.05	0.01*	-15.84	0.33	-232.79	201.12	0.89
Reconstructive surgery	-0.14	-93.30	-224.88	38.27	0.16	4.77	80.00	-138.88	148.42	0.95

CI: confidential interval.

### ROC analysis of age on masticatory function and occlusal force

**Fig. 3:**
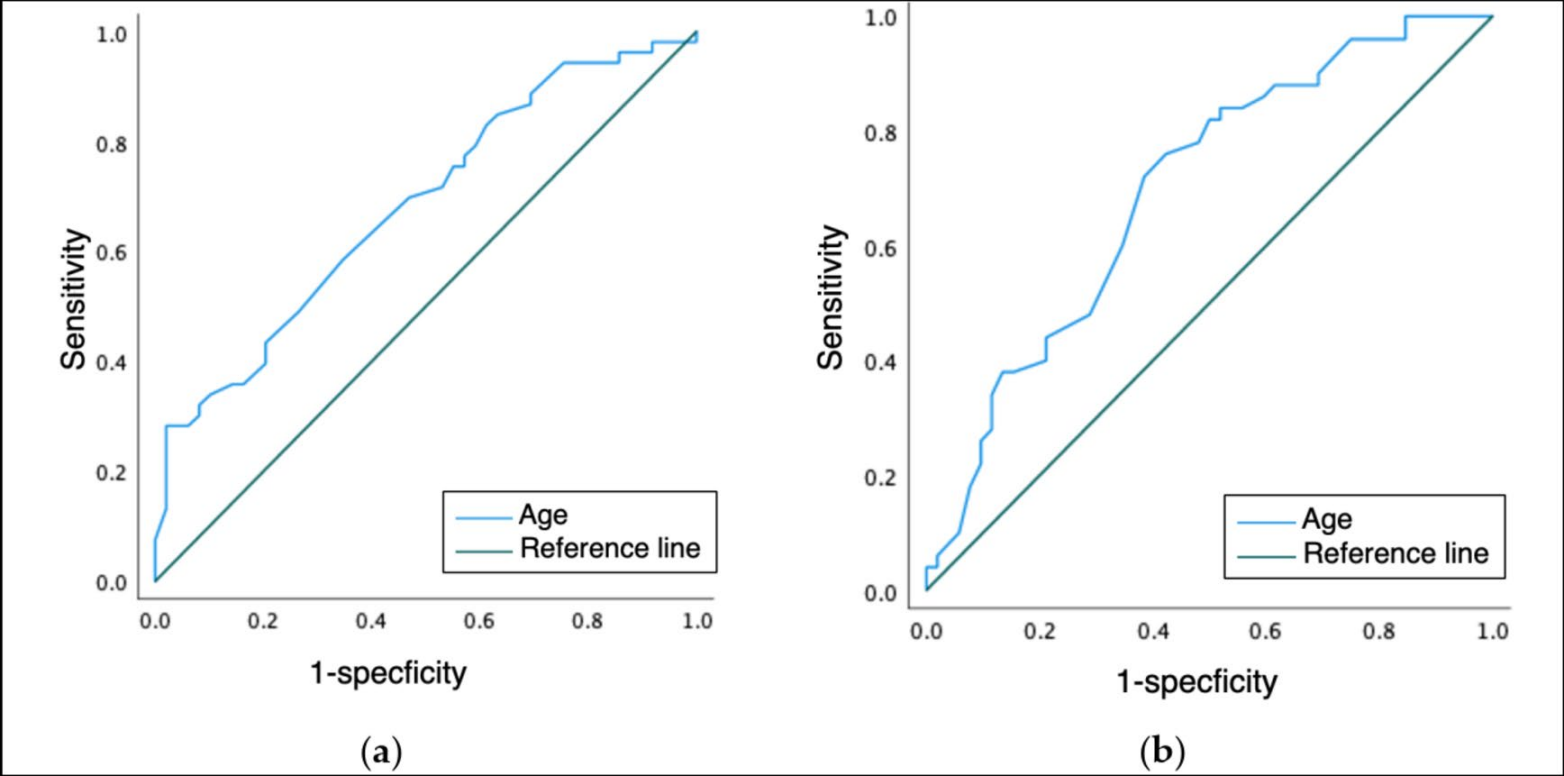
Receiver operating characteristic curves for age and each oral function (a) masticatory function; (b) occlusal force.

For masticatory function, the cutoff age was 84 years when the point of the maximum AUC was 0.68, and the curve was used to determine the cutoff (Fig. 3-a). For occlusal force, the cutoff age was 75 years when the point of maximum AUC was 0.70, and the curve was used to determine the cutoff (Fig. 3-b).

### Calculation of the odds ratio of postoperative oral dysfunction by age (IPTW method)

When the cutoff age was 75 years, the odds ratio for association with masticatory function was 2.49 (*P* = 0.08). The odds ratio indicating an association with occlusal force was 4.32 (*P* = 0.01; Table 4).

**Table 4:** Association between age and each oral function using propensity score CI: confidential interval.

Items	Odds ratio	95% CI		*P*-value
		Lower	Upper	
Model 1 (Objective variable: Masticatory function [mg/dL])				
Age (< 75 years)	2.49	0.89	6.96	0.08
Age (≥ 75 years)	1.00	ref	ref	ref
Model 2 (Objective variable: Occlusal force [N])				
Age (< 75 years)	4.32	1.48	12.55	0.01*
Age (≥ 75 years)	1.00	ref	ref	ref

## Discussion

The age distribution of the enrolled patients with OSCC affected the quality of the study. The median age of this study was 69.6 years, and the oldest patient was 92 years. This patient can be considered an older patient with OSCC relative to the average age of 87 years for women and 81 years for men in Japan; thus, the study design was appropriate^18^.

This study has two major findings. First, age affects the occlusal force and masticatory function after treatment. The components of the occlusal force include the number of remaining teeth, masticatory muscle groups (temporalis, masseter, lateral pterygoid, and medial pterygoid muscles), and their dominant and trigeminal nerve^19^. Muscle strength declines with aging in healthy older adults, as in locomotive syndrome, and without exception in the masticatory muscle groups^20^. In addition, the number of remaining teeth decreases in older adults because of dental caries and periodontal disease^21^. According to Eichner’s classification, those in category C are particularly prone to a decline in occlusal strength^22^. Another report stated that the occlusal force decreases when the number of remaining teeth is less than 19 ^23^. The population in this study included 41 (40.2%) patients with gingival cancer, suggesting that a high probability of tooth loss occurred with gingival cancer resection, which was further reduced by treatment-related factors. Therefore, it is reasonable that age- and treatment-related factors independently influenced the decline of postoperative oral function. The pathological decline in occlusal strength after treatment may lead to an increase in the number of foods that cannot be chewed, resulting in a decrease in food diversity and nutritional deficiencies of certain types^24^. Therefore, decision-making for the treatment method should take into account the possibility that the occlusal strength of older patients with OSCC may decline more than expected after treatment and consider the planning of prosthetic treatment, including the bone-anchored device for wide edentulous areas using implant therapy^25^.

Masticatory function deteriorates over time, representing oral frailty^26^. Mastication is related to occlusal function; however, the major difference is the contribution of oral motor skills to masticatory ability. Mastication requires food to be repeatedly placed on the occlusal support zone by coordinated movements of the tongue, lip, and buccal mucosa^27^. In the process model reported by Palmer et al., mastication function plays an important role in phase 1–2 transport of swallowing and requires higher levels of oral and pharyngeal movements^28^. Mastication involves brain activity in the cerebellum and other areas that send motor commands, as well as in the cingulate motor cortex and other areas involved in the control of delicate forces^29^. The sizes of the cerebellum and cortex decline with age^30,31^. The decline in the physiological function related to the connection of the brain and oral cavity and the organic changes caused by surgery, in addition to these functional changes in older patients with OSCC, are thought to enhance the decline in masticatory function.

Therefore, the reductions in occlusal force and masticatory function should be assessed as reserve strength when treating older patients with OSCC. In other words, when considering occlusal force and masticatory function, age may not be a risk factor for post-treatment oral dysfunction if the only primary site is the palate and the defect is closed by prior flap reconstruction. In addition, the fact that age is not a risk factor for decreased swallowing function but is a risk factor for functional items limited to oral function is one reason for the difference between the pathogeneses of OSCC and head and neck cancers. Therefore, reconstruction of occlusal and masticatory function, including prosthetic treatment and PAP by oral surgeons or prosthodontists, should always be considered as a treatment option, especially for OSCC in older adults.

A reasonable expectation is that there is a significant relationship between the number of oral bacteria and age. The study population included patients who underwent radiotherapy and had radiation-induced xerostomia. Several reports have indicated that radiotherapy affects the salivary glands and decreases saliva production^32^. In addition, aging has been shown to affect the amount and quality of saliva production and its functional decline, and xerostomia and oral dysfunction are associated with an increase in the number of oral bacteria^33–35^. Thus, the association between age and oral bacterial count was a reasonable outcome.

The second major finding was that the cutoff value for age at risk of developing postoperative dysfunction in patients with OSCC was 75 years. A review of the cutoff values for various outcomes in patients with OSCC showed that the overall survival was 65 years^10^. However, 65 years was the cutoff determined for convenience, but to the best of our knowledge, there are no reports on definitions regarding the age of older patients with OSCC. Therefore, the age of 75 years, as calculated in this study, is the first in the world to define older patients with OSCC based on soft outcomes. One recommendation regarding the definition of older adults with cancer in Japan is that there is a reported difference in life expectancy between the upper and lower quartiles of age, and it is important to identify where the patients to be treated fall within this distribution^36^. Since the age of 78 years was the upper quartile cutoff in this study, there is a need to treat younger patients as older when considering the impact of treatment on oral function as a soft outcome. Future prospective cohort studies evaluating survival and oral function over time will be needed to verify whether our age of 75 years is valid for defining older adults with OSCC.

This study has two limitations. First, because this was a cross-sectional study involving postoperative data, it was not possible to estimate the impact of pre-treatment aging on the decline in oral function due to lack of baseline data. Second, the high heterogeneity of the patient groups affects the reliability of the results, since the primary tumor site and the treatment differ from patient to patient. Third, we did not consider the impact on survival rate or quality of life when a cutoff age of 75 years was used, but we did not believe that treating the younger population as older adults would be detrimental to patients. Fourth, the hospital where the study was conducted is a national university hospital (tertiary referral cancer treatment center) located in a rural area of Japan, with many patients residing in mountainous regions. This region also has a particularly high aging rate. The patient population may differ in terms of accessibility to medical care and health literacy compared to urban areas in Japan, and oral function may be poorer than average prior to the onset of oral cancer. Additionally, as similar results may vary across different countries, it is important to consider the possibility that the cutoff value for age may need to be adjusted when evaluating generalizability.

## Conclusions

Age at the time of OSCC treatment is an independent factor related to postoperative oral function (masticatory function and occlusal strength), and the incidence of oral dysfunction is approximately 2–4 times higher in patients aged > 75 years, suggesting the need for caution in selecting treatment methods and managing the postoperative course.

## Data Availability

The data presented in this study are available on request from the corresponding author.
